# Assessing the need for a mental health services reform in Moldova: a situation analysis

**DOI:** 10.1186/s13033-019-0292-9

**Published:** 2019-06-20

**Authors:** Marjonneke de Vetten-Mc Mahon, Laura S. Shields-Zeeman, Ionela Petrea, Niek S. Klazinga

**Affiliations:** 1 Department Trimbos International, Trimbos, Da Costakade 45, Utrecht, The Netherlands; 2 Department Social Medicine, Academic Medical Centre (AMC), University of Amsterdam (UVA), Meibergdreef 9, Amsterdam, The Netherlands

**Keywords:** Mental health, Services reform, Community-based mental health services, Low-and middle-income country, Situational analysis, Needs assessment, Central and eastern Europe, Former Soviet Union

## Abstract

**Background:**

This study describes the Moldovan mental health system and reform needs before and during the initial phase of the MENSANA project (2014–2022) over the period 2007–2017.

**Methods:**

A situation analysis was performed on: (1) the comparative need based on a country comparison using publicly available mental health system data; (2) the normative need based on a document review comparing the Moldovan mental health services structure with the norms of the WHO on the ideal mix of services, and a content analysis of interview and survey data from professionals (n = 93); (3) the felt need based on a content analysis of survey data from service users and carers (n = 52).

**Results:**

The main finding from the comparative analysis is that mental health care remains largely institutionalized with little alternative care options in the community. Moldova has large mental hospitals and a high number of psychiatric beds per 100.000 population (59.8) in comparison with the South-eastern European Health Network and EU15 average in 2014 (47.63 and 36.61). The country also shows an inversion of the ideal mix of services. This points to the potential need for a mental health system reform which was confirmed by the perspectives of the professionals, service users and carers. The majority of respondents favour a mental services reform (82.8% of the professionals and 92.3% of the care recipients) and express numerous issues and reform needs with the most frequently mentioned being the need to: (1) reintegrate service users in society, community and family; (2) deinstitutionalise and implement CBMHS; (3) improve the accessibility and quality of services, and; and 4) address health workforce issues.

**Conclusion:**

All three types of need explored in the situation analysis (e.g. comparative, normative and felt) point towards the necessity to reform the mental health system in Moldova. However, it is emphasized that this will only materialize when underlying socio-economic challenges that both constrain the implementation of community-based mental health services and foster the dependence of people with a mental illness on inpatient services are addressed.

## Background

A well-functioning health care system responds to the need and expectations of the of the population, improves the health of the population, pools funds in a fair way and makes the most efficient use of available resources [1, 2]. With the collapse of the USSR and the independence of Moldova in 1991 it became clear that the inherited Semashko health system^1^ was not able to fulfil these goals. The health care system was characterized by a disproportional large centrally governed health care infrastructure with specialized physicians working in hospitals dominating the provision of care. The system proved to be unaffordable, inefficient and incapable of responding to the emerging challenges of non-communicable diseases, requiring integrated care delivery structures [3–9].

Since 1991 health care reforms in Moldova aimed to make the health care infrastructure more efficient and effective with the merging of parallel systems and the decentralisation of service provision to regionally located and governed institutes that are more embedded in the community [3, 5, 7, 8]. The Moldovan adoption of the Mental Health Declaration for Europe and the Mental Health Action Plan for Europe in 2005 [10], and the development of a national mental health program since 2007 [11, 12] demonstrate the commitment to implement similar reforms in the mental health system. To support the Ministry of Health (MoH) with the implementation, several internationally funded projects have been initiated starting with the ‘Mental Health Project for South-Eastern Europe’ (2002–2006) [13], followed by the ‘Development of Community Mental Health Services System in Moldova’ project (phase 1: 2005–2007, phase 2: 2009–2012), aiming to develop a network of community-based mental health services (CBMHS) in pilot regions [14, 15].

Despite the political commitment to reform the mental health care services, and the initiation of several reform projects over the last 15 years, actual implementation of community-based mental health care services (CBMHS) has been hard to realise in Moldova [7, 14–19]. There are multiple factors that hamper the implementation of CBMHS, one of which has been the lack of a clear vision on how to organise and implement it [15].

In response to these challenges, a broad implementation plan was developed in 2012 [14] focusing on four main objectives to: (1) build the capacity of primary health care workers; (2) develop CBMHS; (3) establish inpatient facilities in local hospitals; (4) improve the quality of care in mental hospitals. In 2014, the MENSANA project started [20], supporting the MoH with the implementation of the reform to realize these objectives, first in four pilot regions (phase 1: 2014–2018) [21] and later nationwide (phase 2: 2018–2022).

To develop a realistic project plan in order to achieve the objectives, an essential step is to perform a situation analysis that maps the existing mental health system with its functional and dysfunctional aspects, defining the context specific mental health services reform needs [15, 22, 23]. As the last thorough situation analysis of the mental health care system in Moldova dates from 2006 [24], this study aims to describe the mental health system in Moldova through a situation analysis to inform on mental health system reform needs before and at the initial stages of the MENSANA project (2007–2017). The outcomes of this study give insight in the baseline situation and reform needs in Moldova and can be informative for mental health care reforms in similar countries.

In this article, ‘need’ is defined as the capacity to benefit from health care [25]. This situation analysis explores the need: (1) compared to other countries (comparative need); (2) compared to norms set by the WHO for the ideal mental health services structure, and from the perspective of professionals providing care (normative need), and; (3) from the perspective of service users and carers receiving care from services targeted by the mental health services reform (felt need) [26].

## Methods

The situation analysis involved mixed methods to inform on the three different types of need including: (1) the comparative need based on a country comparison using publicly available mental health system data; (2) the normative need based on a document review comparing the Moldovan mental health services structure with the norms of the WHO on the ideal mix of services, and a content analysis of interview and survey data from professionals (n = 93), and; (3) the felt need based on a content analysis of survey data from service users and carers (n = 52). With the exploration of three types of need informed by a variety of methods we aimed to triangulate data to reduce the impact of potential bias of each separate method. Table 1 gives an overview of the three types of need and the methodology applied.

**Table 1:** The research aim and methods applied in this situation analysis to inform on the three different types of need

Type of need [26]	Method(s)	Data source(s)	Inclusion and exclusion criteria
(1) Comparative need (gap between what services exist in one area and what services exist in another)	(1) Country comparison between Moldova, the South-Eastern European Health Network (SEEHN) countries and the EU 15 average on mental health system indicators	(1) WHO mental health atlas (2011/2014) [27, 28], WHO Mortality database (2014) [29], WHO Health for all database (2014) [30], Global Health Data Exchange tool (2014) [31] and the study of Krupchanka & Winkler (2016) [32]	Indicators inform on the mental health system and data is available from 2014/2011 from Moldova, (some of) the SEEHN countries and the EU15 average
(2) Normative need (what the expert or professional, administrator or social scientist defines as need)	(2.1) Document review providing an overview of the existing mental health services structure in Moldova in comparison with the norms on the optimal mix of services described by the WHO in the ‘pyramid framework’	(2.1) National policy documents (n = 5), reports (n = 6), international reports (n = 10), and service provision- and usage data from the Moldovan National Health Management Centre (NHMC) from 2014	Documents inform on the mental health services structure in Moldova and are written in English between 2007 and 2015
	(2.2) Content analysis using ‘a priori’ and open coding of interview (n = 23) and qualitative survey data and descriptive analysis of 5-point Likert scale question (n = 70) from professionals involved in the mental health services reform (n = 93)	(2.2) Semi-structured interviews (n = 23) with implementation team members (ITM) (n = 11), health care managers (HCM) (n = 12), and surveys with predominantly open-ended questions among health care practitioners (HCP) (n = 70) collected between May and October 2017	Professionals involved in the reform as ITM, HCM or HCP^1^. ITM if they were part of the international MENSANA project team or the local project implementation unit (PIU). HCM and HCP if they worked in their position for at least 3 months in the pilot districts (Soroca, Orhei, Cimislia and Cahul), or in one of Moldova’s three psychiatric hospitals (Chisinau, Balti and Orhei)
(3) Felt need (what the population feel they need)	(3) Content analysis using ‘a priori’ and open coding of qualitative survey data and descriptive analysis of 5-point Likert scale question from service users and carers who use the services part of the in the mental health services reform (n = 52)	(3) Surveys with predominantly open-ended questions among service users (n = 23) and carers (n = 23) collected in July 2017	Service users and carers older than 18 who received care from community mental health care centres (CMHC’s) in the pilot districts (Soroca, Orhei, Cimislia and Cahul), or in one of Moldova’s three psychiatric hospitals (Chisinau, Balti and Orhei)

The aim of the study is to describe the mental health system in Moldova through a situation analysis to inform on mental health system reform needs before and at the initial stages of the MENSANA project (2007–2017)

^1^HCM—Health care managers in the four pilot districts of the CMHC’s, the primary health centers that house the CMHC’s and the directors of the three psychiatric hospitals in Moldova; HCP—Health care practitioners that provide care in the four pilot districts in the CMHC’s, the primary health centers that house the CMHC’s, and in the three psychiatric hospitals in Moldova including psychiatrists, psychologists, nurses, family doctors, family doctor nurses, psychologists and social assistants

### Comparative need based on a country comparison

The country comparison includes Moldova, the other countries part of the South-eastern Europe Heath Network (SEEHN) [33], and the EU15^2^ average to contrast data from the SEEHN. The SEEHN countries were included because they are similar in terms of development, geopolitical context and they all have been sites for projects aiming to improve health in the region, including mental health [13].

The comparison includes mental health system indicators primarily from the WHO Mental Health Atlas (2011/2014) [27, 28], indicators from other data-bases and the study of Krupchanka and Winkler [32] (Tables 1, 3). Krupchanka and Winkler examined the state of mental healthcare systems in Eastern Europe, including the SEEHN countries Moldova, Bulgaria and Romania. They used data from WHO Mental Health Atlas and calculated change in in-patient care facilities and mental hospital beds between 2011 and 2014. In this study additional data was collected and changes were calculated for the other SEEHN countries.

### Normative need based on WHO norms and the perspective of professionals

**Fig. 1:**
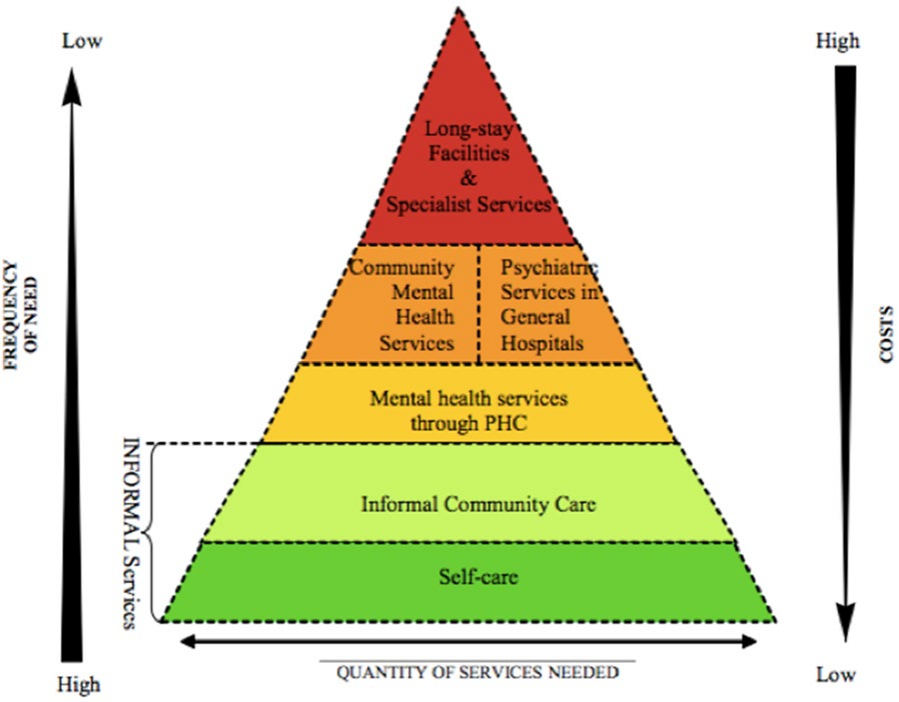
The WHO Pyramid Framework describing the optimal mix of services for mental health [34]

**Fig. 2:**
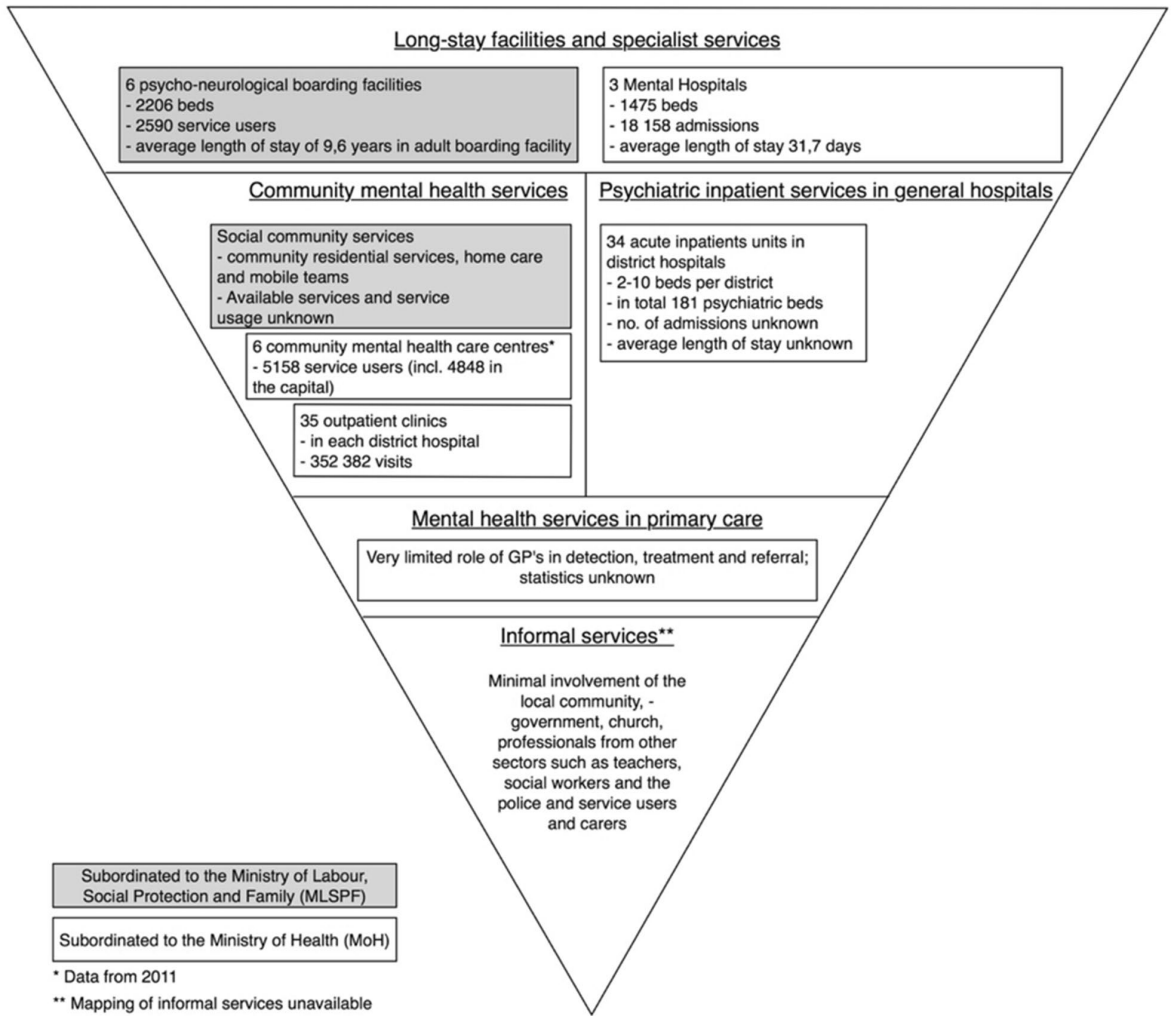
The mix of mental health services in Moldova in 2014

A document review was performed to describe the existing mental health services structure in Moldova in comparison with the ideal structure defined by the WHO in the ‘Pyramid Framework’ (Fig. 1) [34]. An adapted version of the pyramid was developed by the authors to display the services structure in Moldova in 2014 (Fig. 2).

The WHO ‘Pyramid Framework’ stipulates that informal services including community care and self-care should constitute the bulk of care. Self-care refers to self-management with support from carers to prevent and cope with mental illness. Informal community care includes support provided by traditional healers, non-specialized health worker or lay health workers, professionals from other sectors, civil society organizations, and family- and user organisations. When care needs cannot be met at these levels, entry into the formal health system should be via PHC providers who identify patients in early stages of their illness, refer those with severe mental illness to specialist care and manage those who are stable or have a mild- to moderate mental illness. People with severe MH problems might require specialized outpatient care, which includes CMHC’s, rehabilitation services, mobile crisis teams, supervised residential services, and home care. When people have severe MH needs that cannot be resolved in the community, specialized inpatient care may be required, either in in psychiatry wards in general hospitals or psychiatric hospitals. Only a small minority of people, in need of long-term high intensity care, should have access to long stay- and specialist inpatient services [34].

Documents fitting the criteria (Table 1) were retrieved via an internet search and in consultation with MENSANA project implementation team members who had access to policy- and project documents that were not published on the internet. The internet search was done using the following search terms in different order and combination: “mental health”, “mental health system”, “health system”, “health care”, “reform” and “Moldova”. Referred documents that fit the criteria were also included in the analysis.

A content analysis was performed identifying and categorizing information on the mental health services in Moldova in a table structured according to the WHO pyramid framework to allow for comparison between the existing services and the norms set out by the WHO. Data from the documents was supplemented with service provision- and usage data from the Moldovan National Health Management Centre (NHMC) from 2014 [35]. This information was summarized in Fig. 2.

The normative need was additionally informed by the perspective of professionals involved in the mental health services reform through semi-structured interviews (n = 23) and surveys (n = 70) including implementation team members (ITM), health care manager (HCM) and health care practitioners (HCP) (see Tables 1 and 2 for information on methodology and participants). The semi-structured interview guide and the survey with primarily open-ended questions were developed and piloted in collaboration with Moldovan public health researchers to ensure questions and prompts were culturally sensitive and clear.

**Table 2:** Overview of research participants including professionals (normative need), service users and carers (felt need)

	Soroca (%)	Orhei (%)	Cimislia (%)	Cahul (%)	Balti (%)	Chisinau (%)	Total (%)	Male (%)	Average age
Surveys									
(1) Health care practitioners (HCP)									
District HC’s	5	5	4	5		5	24	2	
CMHC’s	7	6	6	4			23	2	
Mental hospitals		3			10	10	23	6	
Total	12	14	10	9	10	15	70 (48.27)	10 (14.28)	44
(2) Service users									
CHMC level	6	3	5	5			19	9	
Mental hospital level		3			3	4	10	4	
Total	6	6	5	5	3	4	29 (20)	13 (44.82)	45
(3) Carers									
CMHC level	4	3	5	2			14	3	
Mental hospital level		2			2	5	9	3	
Total	4	5	5	2	2	5	23 (15.86)	6 (26.08)	53
Total surveys	22	25	20	16	15	24	122 (84.13)	29 (23.77)	47
Interviews									
(4) Implementation team members (ITM)									
International							7	4	
Local							4	1	
Total							11 (7.58)	5 (45.45)	49
(5) Health care managers (HCM)									
District HC’s	1	1	1	1			4	2	
CMHC’s	1	1	1	1			4	2	
Mental hospitals	1				2	1	4	2	
Total	3	2	2	2	2	1	12 (8.27)	6 (50)	47
Total Interviews	6	4	4	4	4	2	23 (15.86)	11 (47.82)	48
Total participants	28 (19.31)	29 (20)	24 (16.55)	20 (13.76)	19 (13.1)	26 (17.93)	145 (100)	40 (27.58)	48

Interviewees were purposively sampled, and survey respondents were randomly sampled on location using a list of the available professionals that day. Interviews were done in English and when this was not possible, they were held in Romanian or Russian with an interpreter who provided simultaneous translation. Surveys were distributed and collected on location in Romanian and Russian.

Interviewees were asked whether they think there was a need for a mental health services reform, and survey respondents were asked through a 5-point Likert scale question whether they agreed with the statement “In Moldova there is a need to implement a reform according to a CBMHS model”. Subsequently they were asked to elaborate their answer, to specify what changes they would like to see and what would be needed to make these changes happen.

The responses to the 5-point Likert scale question were analysed using the ‘document variable statistics’ function in MAXQDA 2018 [36]. The written answers to the open survey questions were translated into English and inserted in MAXQDA software together with the transcriptions of the interviews for content analysis, categorizing reform needs according to a predetermined codebook based on the WHO health system building blocks model [2]. This model describes the essential elements of a well-functioning (mental) health system. With this categorization we could identify the (mental) health system elements that would need most attention according to the respondents. Reform needs identified beyond the mental health care system were labelled with emerging codes. Two researchers coded the data, discussed the outcomes, refined the codebook and recoded the data until agreement was reached on the main reform needs. Subsequently, the reform needs were ranked based on their frequency mentioned (by how many respondents) giving an indication of the importance of the respective need. Finally, differences and commonalities in perceived needs between the stakeholder groups were analysed and reported.

### Felt need based on the perspective of service users and carers

A similar survey was simultaneously developed and distributed to explore the felt need among care recipients of mental health services part of the reform in Moldova (n = 52) including service users (n = 29) and carers (n = 23) (see Tables 1 and 2 for information on methodology and participants).

Service users at the psychiatric hospitals were randomly sampled on location using a list of people present that day. The rest of the respondents were conveniently sampled as they were approached in and around the psychiatric hospital or via their HCP if they received care at a CMHC.

Surveys were distributed and collected on location in Romanian and Russian and if needed respondents were guided through the questions by trained research assistants. They were given the same 5-point Likert scale question as the professionals asking them to specify their answer. Afterwards they were asked whether they had any suggestions to improve care. The responses were analysed in the same way as the data retrieved from the surveys for professionals.

## Results

### Comparative need emerging from a country comparison

With 8.03% DALYs accounted for mental disorders and a prevalence of mental disorders of 17.34% Moldova has the highest reported disease burden in comparison with the other SEEHN countries, but a lower disease burden in comparison with the EU15 average (10.25% and 18.04 respectively). Moldova reported the highest suicide rate per 100.000 population [8, 12] both in comparison with the SEEHN countries and the EU15 average (8.94) in 2014 (Table 3).

**Table 3:** Comparative need emerging from country comparison

	Moldova	Albania	Bosnia and Herzegovina	Bulgaria	Croatia	Macedonia	Monte-negro	Romania	Serbia	SEEHN Average	EU 15 average
General information											
% Disability adjusted life years accounted for mental disorders*	8.03	6.82	6.68	5.06	6.72	5.92	6.99	5.43	5.9	6.39	10.25
% Prevalence mental disorders*	17.34	14.38	15.81	14.79	15.46	14.61	14.76	14.28	14.89	15.15	18.04
Suicide (age-standardized rate per 100,000) **	13.8	NR	6.2	6.9	12.3	6.8	NR	8.8	10.6	9.34	8.94
Existence of mental health policy	Yes	Yes	Yes	Yes ***	Yes	Yes	Yes	Yes	Yes	100%	93%
Implementation status	Partial	Partial	Partial	Partial ***	Partial	Partial	Partial	Partial	Partial	None, 0%; Partial, 100%; full 0%	None, 0%; Partial, 46.6%; full 53.5%
Resources for mental health											
Total health expenditure as % of the GDP (WHO Estimates)****	10.3	5.9	9.6	8.4	7.8	6.5	6.4	5.6	10.4	7.87	9.83
Mental health spending per capita (US$)	4.77	NA	1.89	NR	NA	NA	NA	NA	NA	3.33	293.72
Total no of mental health workers per 100,000	65.2	13.5	23.4	NR	NR	NR	35.2	36.3	21.8	32.57	127.2
No of psychiatrists per 100,000	5.92	1.32	4.00	NR	NR	NR	8.69	5.97	7.35	5.54	14.1
Institutional care											
Total no. of mental hospitals in 2011 (per 100,000)***	3 (0.08)	2 (0.06)	6 (0.16)	12 (0.16)	7 (0.16)	4 (0.2)	3 (0.48)	39 (0.18)	5 (0.05)	9 (0.17)	73.62 (0.27)
Total no. of beds in mental hospitals in 2011 (per 100,000)***	2080 (58.17)	520 (16.41)	467 (12.42)	2705 (36.08)	3353 (76.04)	1150 (56.28)	332 (53.08)	8107 (38.26)	3880 (39.37)	2510.44 (42.90)	11,021.54 (44.72)
No. of beds per mental hospital in 2011 ***	693.3	260	77.83	224.41	479	287.5	110.67	207.87	776	346.40	198.2
Total no. of mental hospitals in 2014 (per 100,000)	3 (0.08)	2 (0.06)	3 (0.08)	NR	8 (0.19)	4 (0.2)	2 (0.32)	35 (0.16)	8 (0.08)	8.13 (0.15)	102 (0.21)
Total no. of beds in mental hospitals in 2014 (per 100,000)	2070 (59.8)	490 (15.4)	NR	NR	3375 (79.0)	NR	261 (42.0)	10 950 (50.6)	3692.67 (39.0)	3473.12 (47.63)	13 373.3 (36.61)
No. of beds per mental hospital in 2014	690	245	NR	NR	421.87	NR	130.5	312.86	461.58	376.97	184.6
Changes (%) in total no. of mental hospitals 2011–2014 (% per 100 000)*****	0% (0%)	0% (0%)	− 50% (− 50%)	NR	14.28% (18.75%)	0% (0%)	− 33.34% (− 33.34%)	− 10.26% (− 11.12%)	60% (60%)	− 9.67% (− 11.77%)	38.5% (− 23.31%)
Changes (%) in total no. of beds in mental hospitals 2014–2011 (% per 100,000)*****	− 0.49% (2.8%)	− 5.77% (− 6.22%)	NR	NR	0.65% (3.89%)	NR	− 21.34% (− 20.88%)	35.06% (32.35%)	− 4.83% (− 0.94%)	38.34% (11.02%)	21.33% (− 18.14%)
Changes (%) in total no. of beds per mental hospital 2014–2011*****	− 0.48%	− 5.77%	NR	NR	− 11.93%	NR	17.91%	50.5%	− 40.52%	8.82%	− 6.8%

SEEHN—South Eastern European Health Network before its enlargement in 2011 with Israel (Albania, Bosnia and Herzegovina, Bulgaria, Croatia, Montenegro, the Republic of Moldova, Romania, Serbia and the former Yugoslav Republic of Macedonia); EU15—15 member states of the European Union before its enlargement in 2004 (Austria, Belgium, Denmark, Finland, France, Germany, Greece, Ireland, Italy, Luxemburg, The Netherlands, Portugal, Spain, Sweden, the UK)

NR, not reported; NA, item not applicable; UN, information unavailable

* Data from the Global Health Data Exchange 2014: http://ghdx.healthdata.org/gbd-results-tool

** Data from WHO mortality database 2014: http://apps.who.int/healthinfo/statistics/mortality/whodpms

*** Data from the WHO Mental Health Atlas 2011: http://www.who.int/mental_health/evidence/atlas/profiles/en/

**** Data from the WHO HFA-DB 2014: https://gateway.euro.who.int/en/datasets/european-health-for-all-database/

***** Indicators and data for Moldova, Bulgaria and Romania from Krupchanka and Winkler [32]

Similar to the other SEEHN countries in 2014, Moldova has a low availability of resources for mental health in comparison with the EU15 average. Despite spending a high percentage (10.3%) of the GDP on health (in contrast with the SEEHN average percentage of 7.87% and the average EU15 percentage of 9.83%), it spent only $4.77 per capita on mental health (compared to 293,72$ per capita in the EU15 average). Moldova had twice the amount of mental health workers per 100,000 population in comparison with the SEEHN average [31, 56], which is still far below the average available mental health care workers in the EU15 (127.2). The number of psychiatrists per 100.000 population in Moldova (5.92) is in line with the SEEHN average [5, 53], but again far below the EU15 average [1, 13].

In 2014 Moldova had a higher number of mental hospital beds per 100,000 population (59.8) and a far higher number of beds per mental hospital (690) than both the SEEHN (47.63 and 376.97) and EU15 average (36.61 and 184.6). The country has shown no decline in number of mental hospitals between 2011 and 2014 in contrast with an average decline in the number of mental hospitals per 100,000 population both in the SEEHN (− 11.77%) and the EU15 countries (− 23.31%). In the same period Moldova showed a small increase in the total number of mental hospital beds per 100,000 population (2.8%) in comparison with a higher increase seen in the SEEHN (11.02% on average), which is in contrast with the deinstitutionalisation trend seen in the EU15 region with an average decline of 18.14%.

### Normative need emerging from a comparison between the existing and ideal mix of services

The Moldovan mix of mental health care services shows an inversion of the WHO ‘Pyramid Framework’ (Fig. 2). In other words, long-stay facilities and specialist services provide the bulk of care, followed by traditional outpatient services, with limited services offered in the community by primary care-, social care- or mental health care professionals. Informal services seem underdeveloped with little to no involvement of community stakeholders.

The MoH and the Ministry of Labour Social Protection and Family (MLSPF) both provide services for people with a mental illness. The MoH provides medical services for people with a mental illness, whereas the MLSPF states responsibility for social services for people with disabilities, including mental disabilities (Fig. 2).

#### Long stay facilities and specialist psychiatric services

The majority of mental health care is provided by the three psychiatric hospitals governed by the MoH [14, 16, 18, 37, 38] with in total 1475 beds and 18.158 admissions in 2014 [35]. The hospitals absorbed 80–85% of the financial resources [14, 37, 38] and 76% of the human resources allocated to mental health [38]. The average length of stay was 31.7 days in 2014 [35], although it must be noted that this number most likely does not reflect reality due to incentives to register a longer or shorter period of stay. Long-term care for people with a mental disability is mainly provided by six institutions (2 for children, 4 for adults), governed by the MLSPF referred to locally as psycho-neurological boarding facilities with, in total, 2206 beds in 2014 [39, 40]. In 2014, 2590 people (79.5% adults) used the services and the average length of stay in the adult boarding facilities was 9.6 years [40]. Care provided in these institutions include social and medical services: treatment with medication; provision of food, clothes and footwear; occupational therapy; and kinetic therapy [7]. Care provided in the psychiatric hospitals and the psycho-neurological boarding facilities are described as being of poor quality based on earlier observations and interviews with service users [16, 37–39]. Treatment practices are referred to as outdated [14, 16, 37, 39] with a strong medical focus and little emphasis on rehabilitation, psychotherapy and recovery [37].

#### Community mental health services

The implementation of CBMHS for mental health is limited both in the mental health system and in the social care sector. The vast majority of mental health care in the community is reported to be provided in 35 traditional outpatient clinics in each district hospital, with 352.382 visits in 2014 [35]. Care provided in these clinics is limited, as home visits are not part of practice, and a typical visit would consist of a basic assessment, a prescription of medication by the psychiatrist [14] or the delivery of a certificate needed for a job application, driving licence or firearm (31% of the visits in 2014) [35]. There were officially 26 CMHC’s subordinated to the MoH in 2014, of which six centres (set up with the help of NGO’s) were functioning in the cities of Balti (n = 2), Chisinau (n = 2), and the districts of Ungheni and Rezina [18, 41]. Services provided in these centres are more extensive and include pharmacotherapy, counselling, day care, temporary shelter, home care, supported housing, legal assistance, occupational therapy and emergency medical care. A limited amount of people made use of these services in 2011; a total of total 5158 of which the majority in the capital Chisinau (n = 4848) [18]. Social CBMHS for people with a mental disability governed by the MLSPF comprise of community residential services, home care and mobile teams [42]. Collaboration between the services provided in the community is reported to be insufficient [18, 37].

#### Psychiatric inpatient services in general hospitals

According to the NHMC, there were 181 psychiatric beds in 34 of the 35 districts in the country, ranging from 2 to 10 beds per district in 2014 [35]. This accounts for 5.22 beds per 100,000 population and is in stark contrast to the EU 15 average of 337.03 acute inpatient beds in the same year [30]. Although the data from the NHMC indicates that acute inpatient wards have been set up around the country, personal communication of the authors with professionals learned that the wards are not functioning in practice due to a lack of dedicated space and staff.

#### Mental health services in PHC

The role of the of primary care doctors and nurses in the detection and treatment of mental illness, and the referral of patients with a severe mental illness is limited [4, 14, 37, 41, 43]. There are several barriers to enhancing the role of family doctors in mental health. First, they are overburdened and reluctant to take up new tasks. In 2014, there were 50,4 general practitioners (GP’s) per 100,000 population (a total of 1746 GP’s in the country) [35], compared with the EU 15 average of 87.25 GP’s per 100,000 population in 2013 [30]. Family doctors are incentivised to take on responsibilities for other prioritized health conditions making it more difficult to spend time on mental health care [14]. Other barriers include their lack of authority to prescribe psychotropic medication [14], their lack of education, knowledge and skills [14, 15, 44]. They also receive insufficient practical guidance to take up their new role, including an implementation plan [15, 17], protocols and specialists supervision [7]. Another important factor is that patients were not aware of, or did not trust, the role of family doctors in mental health care [14, 43].

#### Informal services

Informal services are reported to be underdeveloped in Moldova [37]. The local authorities and community including the church, professionals from other sectors such as teachers, social workers, and the police had minimal involvement in the provision of services and supports [45]. Formal service-user and family organizations are non-existent [15], service users- and carers are not well informed about their rights, and insufficiently involved in the care processes [39]. Stigma, discriminatory behaviour and legislation make it difficult for people with a mental illness to exercise their rights and to receive the support they need to live in the community [37, 39]. Local and international NGO’s reliant on donor funding provide information, assistance and care at the community level (accounting for 1.1% of the total health expenditure in 2010) [7]. Unfortunately, a clear mapping of these services is unavailable [39].

### Normative need emerging from the perspective of professionals

The normative need for a mental health reform was explored through surveys (n = 70) and interviews (n = 23) among professionals (n = 93) including health care practitioners (HCP) (n = 70), implementation team members (ITM) (n = 11) and health care managers (HCM) (n = 12) (Table 2).

**Fig. 3:**
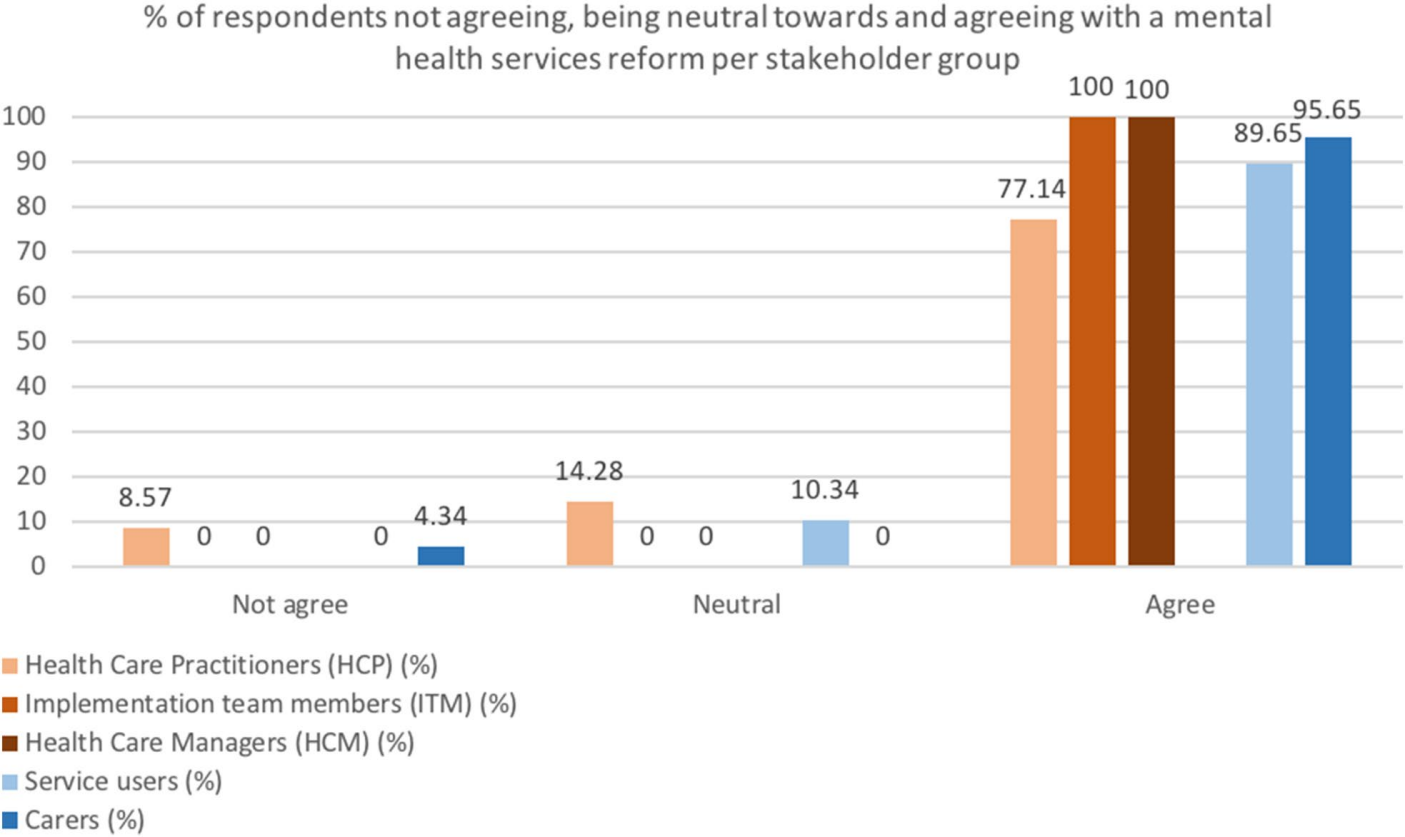
The percentage of respondents including professionals (normative need), service users and carers (felt need) not agreeing, being neutral towards and agreeing with a mental health services reform

The majority of professionals (82.79%) including all the interviewed ITM, HCM, and 77.14% of the surveyed HCP, were in favour of a mental health services reform (Fig. 3). The remaining 22.86% of participants either did not perceive a need for a reform or were indifferent. In the latter group, reasons included the belief that only the mental hospital can provide the continuous supervision needed, patients returning home will create difficulty in the family and community, family and society are not ready for CBMHS, and that medical professionals will lose their job through a services reform. Family doctors and nurses added they did not have time or incentives to take up extra mental health care tasks.

**Fig. 4:**
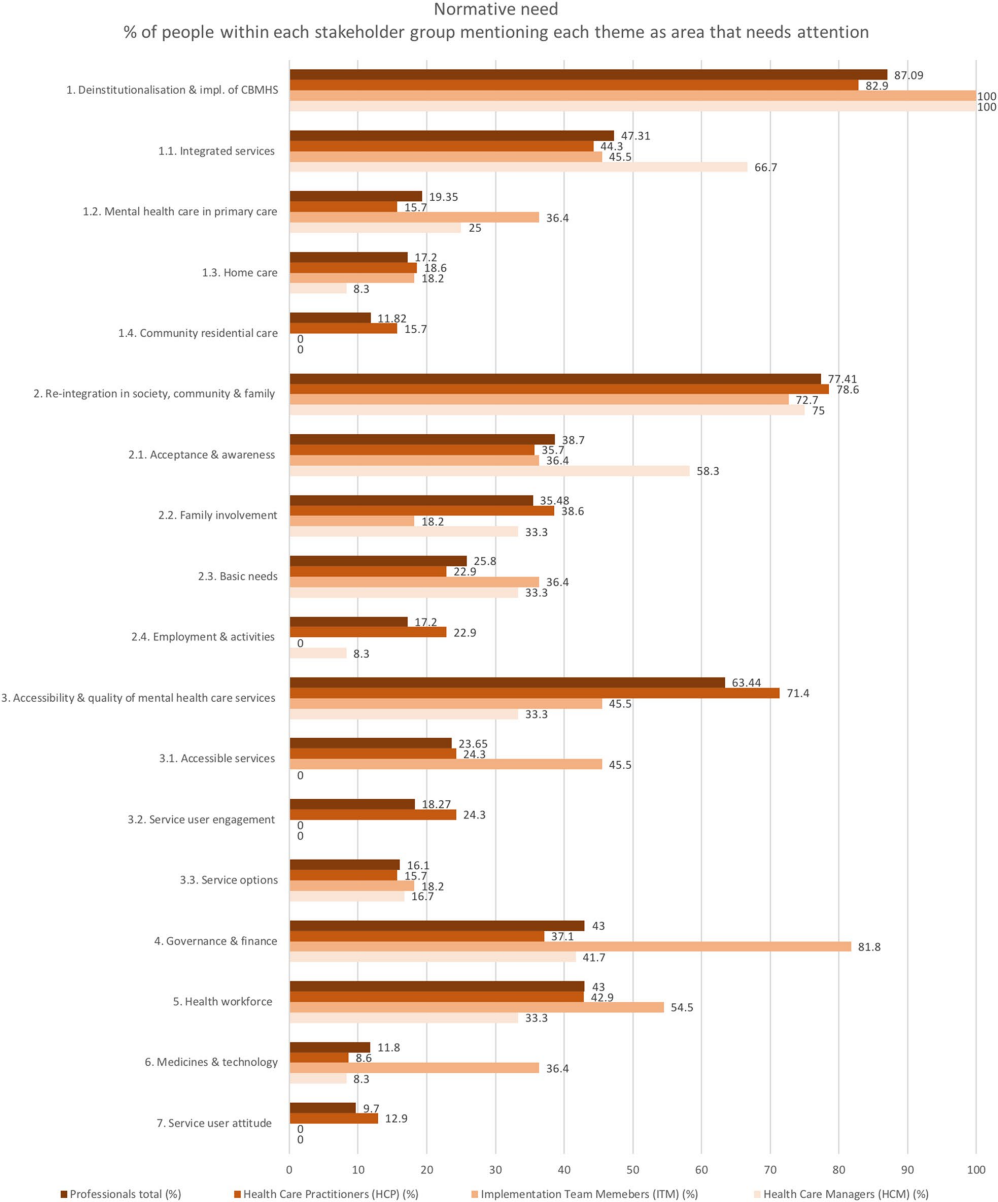
The themes that need to be addressed in the perception of the professionals (normative need) in order of frequency mentioned

A number of issues and reform needs were expressed by the professionals (Fig. 4), with the most mentioned being the need to: (1) deinstitutionalise and implement a CBMHS model with integrated services; (2) reintegrate service users in society, community and family; (3) improve access and quality of services; (4) improve governance and finance; and (5) address health workforce issues.

#### Deinstitutionalisation and implementation of CBMHS

The vast majority of professionals (82.9% of the HCP and all the ITM and HCM) emphasised the need for more alternative mental health services outside the hospital, focussed on the detection of mental illness, prevention of hospitalisation and the reintegration and rehabilitation of service users. They mention that in order to provide sufficient support and prevent hospitalisation diverse medical services, social services, and other sectors such as the police and education should be strengthened and connected:

*ITM 6: “Care was mainly offered in the three main hospitals of Moldova in Orhei, Balti and Chisinau. People were institutionalized for long periods of time and there is no service to continue the care in the community. That’s why that after a longer period in the hospital they relapse and come back in a short time because there is nothing to support them in the community.”*

*HCP 4112: “Cooperation between diverse institutions and social actors will contribute to the multidimensional approach of the beneficiary, a continuation of not only of medical care, but also social and psychological care”.*

#### Re-integration in society, community and family

The majority (78.6% of the HCP, 72.7% of the ITM and 75% of the HCM) felt that reintegration of people with mental health issues into community life is important. Yet many respondents highlight this is difficult to realize in practice because of a lack of medical, social and financial support in the community. They explain that service users often live in isolation without a social support network due to stigma, migration and weak social services. They are often not able to fulfil their basic resource needs with salary or benefits they receive from the local government such as housing, food and heating:

*HCM 11: “People with a mental illness had a lot of different problems and they were marginalized. All problems were more pronounced as result of migration. There are children and parents left who cannot self*-*manage their money and properties and were at risk of being deprived from what they had.”*

*HCM 12: “The mental health patients were institutionalized so they spent most of their time in the hospital and nobody wanted to deal with them at home. After hospitalization they went nowhere. Taking into account the difficult economic situation in the country and the attitude of the local government they were in a very poor position (…) Frankly speaking they were not considered as human beings. They were considered as a burden to the society, as if the society did not need them.”*

*HCP 4262: “(It is needed) to be involved in the beneficiary’s problems such as their living conditions, family and work place”.*

#### Accessibility and quality of services

The third most mentioned theme by professionals is access and quality of services (HCP 71.4%, ITM 45.5%, and HCM 33.3%). Both HCP and ITM pointed towards the lack of access to services for people living in the rural areas due to the large distances, bad road conditions and the travel costs. Mental health care services are concentrated in the district centres and the cities in the north of the country. All three stakeholder groups mentioned that there is a need for more specialized care where service users can receive timely psychotherapy, occupational therapy, ergo therapy and legal assistance to avoid hospitalisation. HCP’s emphasize that it is important to have time for consultation with the service users to inform, advise and encourage them to obtain better results. ITM’s particularly highlighted the old-fashioned pharmacological treatment and rundown facilities in the mental hospitals as a need for change:

*ITM 1: “Care was very traditional old fashioned, not very friendly towards the patients. (…) Services provided were very basic, primarily medication with occasionally some psychotherapy on private basis. (…) There were problems all around. I would say that the services existed, it’s just that they were heavily institutionalized and heavily medicalized.”*

#### Governance and finance

The fourth most mentioned theme by professionals is governance and finance (HCP 37.1%, ITM 81.8% and 41.7% of the HCM). Respondents commented on the top-down governance style, and inadequate coordination. They also refer to insufficient collaboration between medical and social services, and the lack of clarity among service providers on the treatment and referral of psychiatric, neurologic and addicted patients. ITM and HCM pointed towards the need to adapt legislation for the CBMHS model to function. Some HCM highlighted the need for change of discriminatory legislation that inhibits registered patients from applying for a job or driving licence. All three stakeholder groups pointed to inadequate allocation of resources and the overall lack of funding for treatment and human resources:

*ITM 4: “In Moldova the government is responsible for buying medication. They buy a bulk amount and distribute it to the clinics. They have to use that, even if they don’t need to. The government bought a lot of lithium and the expert told me that nobody knew how to use it. Which means that it wasn’t used and as a consequence the government didn’t buy it anymore. So now there is no lithium available in Moldova to treat bipolar patients. Of course, you can argue there are other medications that can be used to do the job, but they won’t be “state of the art”. It also illustrates that the mental health system it is still organized very top down, and the people who are responsible for the decisions, the policymakers and the decision*-*makers, are often not that medically well informed.”*

#### Health workforce

Related to governance and finance, the majority of the professionals also mentioned the health workforce as an area that needs attention (HCP 42.9%, ITM 54.5% and HCM 33.3%). They argue that existing and additional work forces should be trained to fill the knowledge gap and to make sure that there are sufficient specialists and primary health care workers to work with people with a mental illness, especially in the rural areas:

*HCM 9: “Staff problem remain. (…) It is a problem not only faced by the CMHC’s but also by the medical institutions. This problem is different in each institution. One institution faces the shortage of doctors, another institution has a shortage of nurses”.*

### Felt need emerging from the perspective of service users and cares

Service users (n = 29) and cares (n = 23) (Table 2) provided insight in what care they want and need. In line with the professionals, almost all care recipients (92.3%) including service users (89.65%) and carers (95.65%) were in favour of a mental health services reform (Fig. 3). One service user and carer, both of whom were receiving care from the mental hospital, responded neutrally or did not see the need for reform. They elaborated that, although it was not perfect, the conditions were good in the mental hospital.

**Fig. 5:**
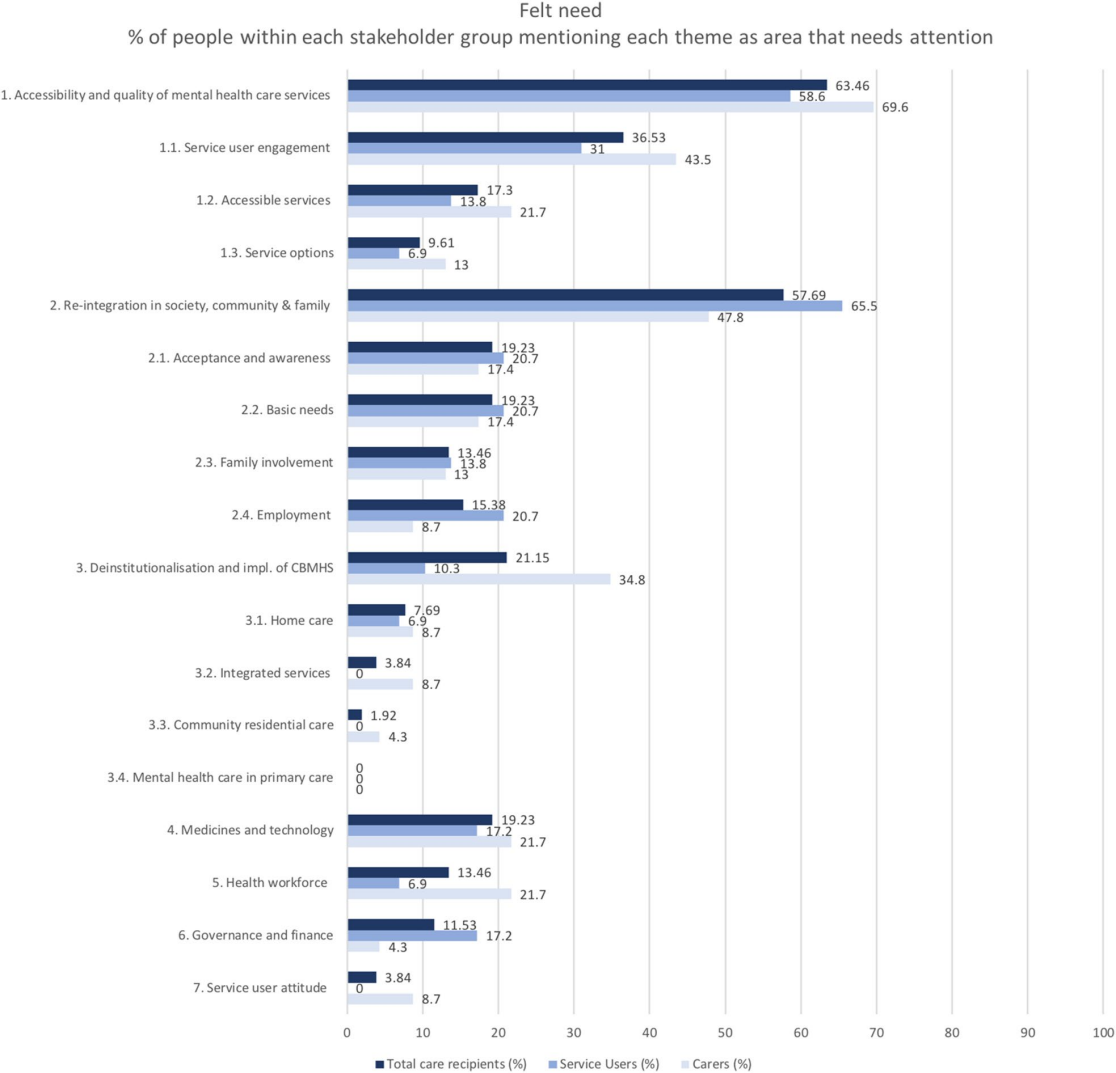
The themes that need to be addressed in the perception of care recipients including service users and carers (felt need) in order of frequency mentioned

A number of issues and reform needs were expressed largely in line with the responses of the professionals, but in a different order based on their frequency mentioned (Fig. 5), with the need to: (1) improve the access and quality of services; (2) reintegrate in society, community and family; (3) deinstitutionalise and implement CBMHS; (4) address problems with medicines and technology and (5) address health workforce issues.

#### Accessibility and quality of services

The most frequently mentioned theme among service users (58.6%) and carers (69.6%), as opposed to the third most mentioned theme among professionals, is the accessibility and quality of services. Long waiting times, travel distance and costs are mentioned as barriers to access care. Respondents express the need for more specialist treatment such as occupational-, kino- and speech therapy. Hospital care is by some referred to as a place where they receive good care, while others call it a prison. Service users and carers receiving care from a community mental health care centre (CMHC) highlight the need for service user engagement including consultation, emotional support, advice and information. Service users express that they are happy with the contact with peers and professionals at the CMHC’s, whereas carers stress the need for more engagement:

*Service user 7141: “There should be community services because when you feel depressed there should be someone who listens to you, encourages you and helps you.”*

*Service user 7251: “Of course it is better to stay at home without problems with her son, so she does not have to stay in the hospital. The conditions in hospital are very good, they feed them well, they take them out for walks, the attitude of the doctors and nurses is very good.”*

#### Reintegration in society, community and family

In line with the professionals, reintegration was the second most mentioned theme for 65.5% of the service users and 47.8% of the carers. Respondents expressed basic needs such as health, family contact, shelter, food, warmth and employment. Some respondents voice that there is no support for them in the community. Service users and carers who receive care from CMHC’s appreciate the emotional and financial support they receive, and stress that employment or participation in other daily activities for service users is important to be part of social life:

*Carer 8124: “People think they will get sick from her daughter. If people come to visit them (at home), they will be rewarded well”.*

*Carer 8142: “There should be the possibility to have a workplace for patients”.*

#### Deinstitutionalisation and implementation of CBMHS

As opposed to the most important theme for professionals, deinstitutionalisation and implementation of CBMHC is the third most mentioned theme among service users (10.3%) and carers (34.8%). The respondents expressed the need for care at, or closer to home to prevent worsening of the situation and hospitalisation:

*Carer 8272: “It would be perfect if ambulatory treatment (at home) will be developed because not every case of mental illness needs to be hospitalized. Periodic follow*-*up of the patient would prevent from worsening situation that leads to hospitalization.”*

#### Medicines and technology

Both service users (17.2%) and carers (21.7%) communicated that they would like (better) medication. This theme was not mentioned by professionals that often:

*Carer 8251: “They should have the last generation equipment and medicines.”*

#### Health workforce

In line with the professionals, the health workforce is the fifth most mentioned theme among service users (6.9%) and carers (21.7%), referring to the need for more doctors and the improvement of their attitude:

*Carer 8265: “The attitude should change. They should understand that the relatives are not their patients. They (the doctors), consider that only they are right. Also, we don’t have always enough money to give them.”*

### Overview results comparative, normative and felt need

The three types of need reinforce each other and sketch a Moldovan mental health care system that remains largely institutionalized and with little quality care options in the community. Both professionals and care recipients are largely in favour of a mental health services reform, and they both express the need to improve formal and informal support in the community to enable people with a mental illness to reintegrate and recover. The difference between professionals and care recipients is that the first group puts more emphasis on the need to address issues related to governance and finance of the mental health system, while care recipients highlight the need to improve access to a greater variety of services and quality medication. Table 4 provides an overview of the results on each type of need.

**Table 4:** Overview results comparative, normative and felt need

Type of need [26]	Informed by	Main outcomes
(1) Comparative need (gap between what services exist in one area and what services exist in another)	(1) Country comparison	Mental health care remains largely institutionalized evidenced by a far higher number of beds per mental hospital (690) and a higher number of mental hospital beds per 100,000 population (59.8) in 2014 than both the SEEHN (376.97 and 47.63) and EU15 average (184.6 and 36.6). In contrast with an average decline of the number of mental hospitals per 100.000 population both in the SEEHN (− 11.77%) and the EU15 countries (− 23.31%) Moldova has shown no decline in number of mental hospitals between 2011 and 2014
(2) Normative need (what the expert or professional, administrator or social scientist defines as need)	(2.1) Comparison Moldovan mental health services structure with norms WHO	The Moldovan mental health services structure shows an inversion of the WHO ‘Pyramid Framework’. In other words, long-stay facilities and specialist services provide the bulk of care, followed by traditional outpatient services, with limited services offered in the community by primary care-, social care- or mental health care professionals. Informal services seem underdeveloped with little to no involvement of community stakeholders
	(2.2) Perspective of professionals involved in the reform including health care practitioners, health care managers and implementation team members	The majority of professionals (82.8%) were in favour of a mental health services reform. A number of issues and reform needs were expressed by the professionals with the most mentioned being the need to (1) deinstitutionalise and implement a CBMHS model with integrated services; (2) reintegrate service users in society, community and family; (3) improve access and quality of services; (4) improve governance and finance; and (5) address health workforce issues
(3) Felt need (what the population feel they need)	(3) Perspective of care recipients of services involved in the reform including service users and carers	Almost all care recipients (92.3%) were in favour of a mental health services reform. A number of issues and reform needs were expressed largely in line with the responses of the professionals, but in a different order based on their frequency mentioned with the need to (1) improve the access and quality of services; (2) reintegrate in society, community and family; (3) deinstitutionalise and implement CBMHS; (4) address problems with medicines and technology and (5) address health workforce issues

The aim of the study is to describe the mental health system in Moldova through a situation analysis to inform on mental health system reform needs

## Discussion

This study aimed to identify the mental health system reform needs through a situation analysis of the Moldovan mental health system before and during the initial phase of the MENSANA reform project (2007–2017). The authors looked at the comparative need (based on a comparison between mental health system indicators from Moldova, countries in the SEEHN and the EU15 average), the normative need (based on a comparison between the Moldovan mental health services structure and norms of the WHO, and the perspective of professionals involved in the reform), and felt need (based on the perception of service users and carers who received care from services targeted by the reform).

The main finding from the comparative need is that mental health care remains largely institutionalized. This is evidenced by the large mental hospitals, the high number of psychiatric beds per 100,000 population, and the absence of a decline in the number of mental hospitals between 2011 and 2014, in contrast with SEEHN countries and the EU15 average. The normative need, informed by the comparison between the existing services structure and the norms set by the WHO supports this finding, showing an inversion of the ideal mix of services in Moldova. Specialist inpatient services and the traditional outpatient clinics provide the bulk of care, with little alternative care options in the community.

This points to the potential need for a mental health system reform, which was underlined by the normative and felt need expressed by key stakeholders. The majority of respondents favour a mental services reform (82.8% of the professionals and 92.3% of the care recipients), expressing numerous issues and reform needs, with the most frequently mentioned the need to: (1) reintegrate service users in society, community and family; (2) deinstitutionalise and implement CBMHS; (3) improve the accessibility and quality of services, and; (4) address health workforce issues.

All three perspectives indicate the ongoing need for the strengthening and implementation of CBMHS to care for people with a mental illness in the community. However, it is emphasized that social and financial support structures should be in place involving the local authorities, social services, family and community stakeholders to enable service users to live, reintegrate and recover in the community. In order to set up such multilateral support network, the findings suggest that underlying socio-economic challenges that both constrain the implementation of community-based mental health services and foster the dependence of people with a mental illness on inpatient services should be addressed.

These findings mirror the situation in other Eastern European and former Soviet countries where the shift towards CBMHS is desired by service users [46], reflected in policy aims and reform efforts, but has limited results in practice [8, 23, 32, 46–50]. Mental health system reforms often do not succeed in in the region due to a lack of financial and competent human resources [8, 32, 48–50]. Mental Health systems are often underfunded by the government, and reform activities, including training of human resources, rely on support of international organizations [8, 48, 50]. Working in mental health is not popular because of stigma and undesirable working conditions. Moreover, many of those trained in low- and middle income countries in the region leave as they have better prospects in other Russian speaking countries where they are offered a higher salary and quality of life [8, 50].

In addition, the scarce available resources are not always adequately allocated [23, 51, 52]. Local economic, epidemiological, social studies and monitoring and evaluation activities are rarely funded or performed, resulting in unrealistic policies and plans, and non-transparent decision-making [8, 50].

Socio-economic challenges in countries in the region do not only constrain mental health service planning and provision, but also foster the demand for inpatient hospital care. Employed middle class people struggle to provide for their basic needs. The situation for people with a mental illness is even more challenging due to stigma, discrimination, isolation, homelessness, unemployment and the absence of a social security system [46, 48, 50, 53]. Some of these people turn to inpatient hospital care as an alternative for community social services and housing [8, 50]. This problem is difficult to address since it is hard to convince state authorities to invest scarce resources in housing and financial support for people with a mental illness while many people deal with similar problems [8].

The MENSANA project phase 1, operating according to the implementation plan developed in 2012 [14] responds partly to the reform needs and challenges identified in this study. With the funding and implementation of a CBMHS model in four pilot districts setting up CMHC’s, local acute inpatients units, involving primary health care practices and mental hospitals the need for medical support in the community is addressed. However, to materialize the ongoing implementation of CBMHS and the reintegration and recovery of service users in the community, the underlying socio-economic challenges should be given more attention.

Future mental health system reform projects in Moldova and in the region should have a broader approach to address the lack of, and often inadequate allocation of financial and human resources. At the same time service users should have access to financial and social support to help them reintegrate and recover in the community. Recommendations for action include human resource development and retention [54], research capacity building and the implementation of more studies, monitoring and evaluation activities at local level to inform decision-making and to secure structural funding [32, 46, 50]. Other strategies include the involvement of service users [32, 50], families, professionals, community stakeholders and social services in the development of mental health policies and services to improve financial and social support for service users in the community.

### Strengths and limitations

This study contributes to the scarce available literature on mental health services reform needs in Eastern Europe, and the even scarcer available literature on country specific reform needs in the region [47]. The strength of this study is the triangulation of three different data sources. However, each research method also has its limitations. The country comparison is based on publicly available data reported by local health experts, not always accurately reflecting the situation in practice [55]. Information on some indicators was available for only a few countries making a fair comparison difficult. In addition, the definition of indicators such as the number of mental hospital beds could be interpreted differently per country. Additionally, the document analysis only included documents in English, possibly missing important information from local documents. Lastly, the majority of the research participants were involved in the reform and this might have resulted in biased responses in favour of the reform. Local managers and many of the professionals were trained as part of the reform, and most service users and carers received care from CMHC’s part of the new developed CBMHS.

## Conclusion

All three perspectives on need explored in this situation analysis (e.g. the comparative, normative and felt need) concur towards the necessity for a mental health system reform in Moldova according to the CBMHS model. However, it is emphasized that these will only materialize if underlying socio-economic challenges that both constrain the implementation of CBMHS and foster the dependence on inpatient hospital care are addressed. In executing the MENSANA project these findings are taken into account, and alongside service delivery redesign, emphasis is put on the broader agenda of informed decision-making, human resource development and retention, as well as anti-stigma awareness raising activities involving the community.

## Data Availability

Data sharing is not applicable to this article as no datasets were generated or analysed during the current study. Anonymized qualitative data can be obtained on reasonable request from the corresponding author.

## Abbreviations

CBMHS: community-based mental health services
CMHC: community mental health centre
GP: general practitioner
HCM: health care manager
HCP: health care practitioners
ITM: implementation team member
MLSPF: Ministry of Labour Social Protection and Family
MoH: Ministry of Health
NHMC: National Health Management Centre
PUI: Project Implementation Unit
SEEHN: South-Eastern Europe Health Network

## Footnotes

1 The Semashko model is one of the three families of European health care systems in countries that were under the soviet sphere of influence. The model is highly centralized, funded by state budget and intends to guarantee free access to health care for all [56].

2 15 member states of the European Union before its enlargement in 2004 including Austria, Belgium, Denmark, Finland, France, Germany, Greece, Ireland, Italy, Luxemburg, The Netherlands, Portugal, Spain, Sweden, the UK.
